# Tumor‐Specific Drug Release and Reactive Oxygen Species Generation for Cancer Chemo/Chemodynamic Combination Therapy

**DOI:** 10.1002/advs.201801986

**Published:** 2019-01-18

**Authors:** Sheng Wang, Zhantong Wang, Guocan Yu, Zijian Zhou, Orit Jacobson, Yijing Liu, Ying Ma, Fuwu Zhang, Zhi‐Yi Chen, Xiaoyuan Chen

**Affiliations:** ^1^ Department of Ultrasound Medicine Laboratory of Ultrasound Molecular Imaging The Third Affiliated Hospital of Guangzhou Medical University Guangzhou 510150 China; ^2^ Laboratory of Molecular Imaging and Nanomedicine National Institute of Biomedical Imaging and Bioengineering National Institutes of Health Bethesda MD 20892 USA

**Keywords:** Fenton reaction, nanomedicines, polyprodrugs, reactive oxygen species, triggered drug release

## Abstract

The combination of chemotherapeutic drugs and reactive oxygen species (ROS) is a promising strategy to achieve improved anticancer effect. Herein, a nanomedicine (LaCIONPs) that can achieve tumor‐specific chemotherapeutic drug release and ROS generation is developed for cancer chemo/chemodynamic combination therapy. The LaCIONPs are constructed by encapsulation of iron oxide nanoparticles (IONPs) and β‐lapachone (La) in nanostructure assembled by hydrogen peroxide (H_2_O_2_)‐responsive polyprodrug and pH‐responsive polymer. Through the enhanced permeability and retention effect, the nanosized LaCIONPs can accumulate in tumor tissue. After the LaCIONPs are internalized by tumor cells, the structure of LaCIONPs is disintegrated in acidic intracellular environment, leading to rapid release of La and iron ions. Then the released La generates massive H_2_O_2_ through tumor specific catalysis. On the one hand, H_2_O_2_ further reacts with iron ions to produce highly toxic hydroxyl radicals for chemodynamic therapy. On the other hand, H_2_O_2_ also activates the release of camptothecin from the polyprodrug for chemotherapy. The potent antitumor effect of the LaCIONPs is demonstrated by both in vitro and in vivo results. Therefore, the LaCIONP is a promising nanomedicine for tumor‐specific chemo/chemodynamic combination therapy.

Nanocarrier‐mediated chemotherapy has been actively pursued to improve bioavailability of therapeutic drugs and reduce adverse effects to normal tissues.^1^ To date, most of the nanoscale drug delivery systems still face many challenges such as low loading stability and burst drug release, leading to unsatisfactory treatment effect.^2^ Stimuli‐responsive nanomedicines, which are able to realize triggered responses, have been exploited for on‐demand drug release.^3^ Particularly, polyprodrug amphiphiles have been recognized as a promising strategy.^4^ Therapeutic drugs are conjugated to amphiphilic polymers through stimuli‐responsive linkers such as hydrazone bond and disulfide bond, the resulting polyprodrug amphiphiles can self‐assemble into nanomedicines with distinct advantages, such as high loading content, high stability, and prolonged blood circulation.^5^ Upon certain stimulus (e.g., pH, glutathione, enzymes) in tumor microenvironment, therapeutic drugs in their active form will be released due to the cleavage of linkers. However, the generally used pH‐ and glutathione‐responsive drug delivery systems usually suffer from limited selectivity because acidic endosome/lysosome and high intracellular reduction environments are present in both cancer and normal cells. Compared with acidic pH and glutathione, hydrogen peroxide (H_2_O_2_)‐triggered drug release shows higher tumor specificity because the H_2_O_2_ level in normal tissues is much lower than that in cancer.^6^

Reactive oxygen species (ROS), such as hydroxyl radical (·OH) and singlet oxygen, can cause cell death through oxidative damage of lipids, proteins, and DNA.^7^ Furthermore, ROS can also be used to enhance the treatment efficacy of chemotherapeutic drugs, resulting in synergistic cancer therapy.^8^ In recent years, various ROS generation strategies, such as photodynamic therapy, sonodynamic therapy, and chemodynamic therapy, have been developed for cancer treatment.^9^ For example, Fenton reaction utilizes iron‐mediated catalytic reaction to convert less reactive H_2_O_2_ into highly reactive ·OH, resulting in escalated ROS stress inside cells.^10^ Therefore, the combination of H_2_O_2_‐triggered drug release and H_2_O_2_‐involved Fenton reaction is a promising strategy to achieve tumor‐specific chemo/chemodynamic combination therapy.

Although cancer cells have higher H_2_O_2_ concentration than normal cells, the endogenous H_2_O_2_ is still not sufficient for effective reactions. In order to amplify the H_2_O_2_ level, various H_2_O_2_‐generating agents (e.g., cisplatin, glucose oxidase, vitamin C, and their derivatives) were exploited to produce H_2_O_2_ in cells.^11^ Among these agents, β‐lapachone (La), which can generate H_2_O_2_ through the catalysis of the nicotinamide adenine dinucleotide (phosphate) (NAD(P)H): quinone oxidoreductase 1 (NQO1), shows great potential for tumor‐specific H_2_O_2_ level amplification.^12^ Compared to the other H_2_O_2_‐generating agents, La exhibits two main features: first, La shows high selectivity for tumor cells over normal cells due to the overexpression of NQO1 in cancer cells; secondly, La undergoes fertile redox cycles, resulting in rapid and massive generation of H_2_O_2_.^13^

Herein, we developed a nanomedicine to achieve tumor‐specific drug release and ROS generation for chemo/chemodynamic combination therapy (**Figure** **1**). Iron oxide nanoparticles (IONPs) were first prepared and modified with pH‐responsive poly diisopropylaminoethyl methacrylate (PDPA). Then the PDPA‐IONPs and La were encapsulated into nanostructures formed by H_2_O_2_‐responsive poly(ethylene glycol) (PEG)‐poly camptothecin (CPT) prodrug (PEG‐PCPT) and PEG‐PDPA, obtaining the pH‐ and H_2_O_2_‐dual‐responsive nanomedicine (denoted as LaCIONPs). Through the enhanced permeability and retention (EPR) effect, the nanosized LaCIONPs can accumulate in tumor tissues. Once the LaCIONPs enter cancer cells through endocytosis, their structure will be disintegrated under acidic endosome/lysosome environments due to the hydrophobic‐to‐hydrophilic conversion of PDPA, resulting in rapid release of La and iron ions. Catalyzed by NAD(P)H: NQO1, the La generates H_2_O_2_, leading to amplified intracellular ROS level. Then the generated H_2_O_2_ will further react with iron ions through Fenton reaction to produce highly toxic ·OH, resulting in improved antitumor activity. Moreover, the H_2_O_2_ can also cause the cleavage of peroxalate ester linkages and trigger CPT release from the polyprodrug. Owing to the low NQO1 expression in normal cells, the LaCIONPs will not cause significant H_2_O_2_ level amplification and severe side effects in normal tissues. Therefore, this nanomedicine is a promising agent for tumor‐specific chemo/chemodynamic combination therapy.

**Figure 1:**
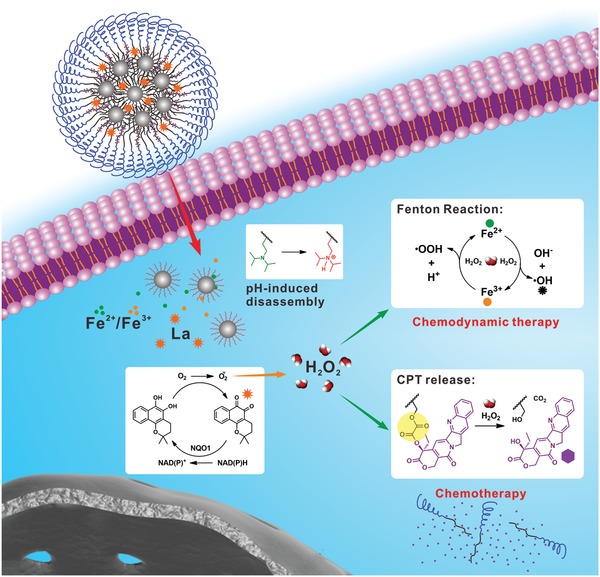
Schematic illustration of LaCIONPs for tumor‐specific chemo/chemodynamic combination therapy.

To prepare the H_2_O_2_‐responsive polyprodrug, the CPT prodrug monomer with a peroxalate ester linkage (CPTMA) was first synthesized (Scheme S1, Supporting Information).[[qv: 4e]] NMR and LC–MS analysis were used to confirm the chemical structure of CPTMA (Figures S1–S3, Supporting Information). Through reversible addition fragmentation chain transfer polymerization, the H_2_O_2_‐responsive polyprodrug PEG‐PCPT and pH‐responsive PEG‐PDPA with a hydrophobic‐to‐hydrophilic transition pH of about 6.3 were synthesized (Schemes S2 and S3, Supporting Information).^14^ The structures of the synthesized polymers were characterized by ^1^H NMR (Figures S4 and S5, Supporting Information). The synthesized PEG‐PCPT showed the typical peak of CPT (Figure S6, Supporting Information). According to the absorbance, the CPT content of PEG‐PCPT was determined to be 55.4%, which was consistent with what was calculated from the NMR result (52.9%). Then an atom transfer radical polymerization (ATRP) initiator was successfully synthesized according to a previous report (Scheme S4 and Figures S7 and S8, Supporting Information).^15^ The phosphate terminated PDPA (P‐PDPA) was synthesized by ATRP (Scheme S5 and Figure S9, Supporting Information). Afterward, IONPs with metastable mixed Wüstite–magnetite (FeO–Fe_3_O_4_) phases were synthesized and modified with P‐PDPA.[[qv: 9e]] The obtained PDPA‐IONPs showed pH‐responsive property: at pH 7.4, the PDPA was a hydrophobic polymer, thus, the PDPA‐IONPs form large aggregates in aqueous solution; however, at pH 5.5, the PDPA became hydrophilic and the PDPA‐IONPs were well dispersed in aqueous solution (Figure S10, Supporting Information). To serve as a control group, upconversion nanoparticles (UCNPs) with a diameter similar to the IONPs were synthesized and modified with P‐PDPA, obtaining PDPA‐UCNPs (Figure S11, Supporting Information).^16^

Then the LaCIONPs were prepared as shown in the schematic illustration (Figure S12, Supporting Information). The PDPA‐IONPs and La were encapsulated into the hydrophobic core of the nanoparticle formed by PEG‐PCPT and PEG‐PDPA. The control groups, LaCUCNPs, LaDIONPs, and LaDUCNPs were also prepared (Table S1, Supporting Information). To evaluate the pH‐responsiveness of LaCIONPs (**Figure** **2**a), the size and morphology of LaCIONPs at different pH values were detected by transmission electron microscopy. As shown in Figure 2b, PDPA‐IONPs were assembled to form LaCIONPs with a diameter of about 150 nm. Dynamic light scattering (DLS) measurement demonstrated that the hydrodynamic diameter and zeta potential of LaCIONPs were 171.4 ± 33.2 nm and –7.5 ± 1.8 mV, respectively (Figures 2d and e). The nanosized diameter and neutral surface charge of LaCIONPs make them suitable for tumor accumulation through EPR effect. However, in an acidic environment (pH 5.5), the LaCIONPs disassembled into independent IONPs with a hydrodynamic diameter of 48.6 ± 9.3 nm (Figures 2c and d). Moreover, the disassembled particles showed positive zeta potential due to the modification of protonated PDPA (Figure 2e). All of these results demonstrated that the LaCIONPs can achieve pH‐responsive disassembly. The control group (LaCUCNPs) showed similar hydrodynamic diameter and pH‐responsiveness (Figures S13 and S14, Supporting Information).

**Figure 2:**
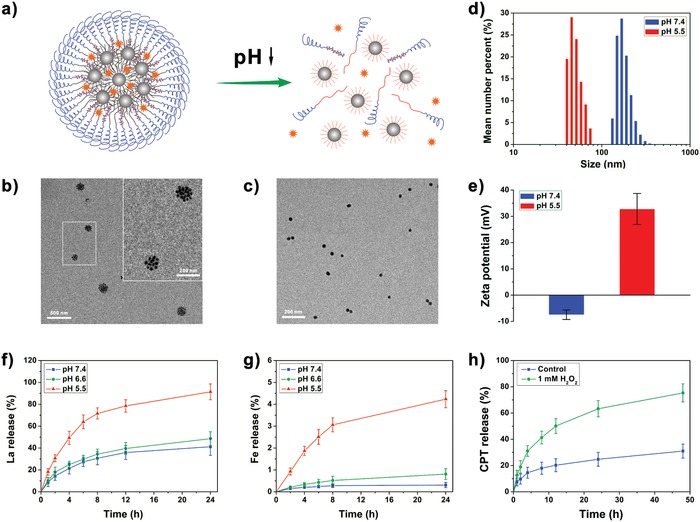
a) Schematic illustration of the pH‐responsive disassembly of LaCIONPs. b) Transmission electron microscopy image of LaCIONPs at pH 7.4. c) Transmission electron microscopy image of disassembled LaCIONPs at pH 5.5. d) Hydrodynamic diameters of LaCIONPs at different pH. e) pH‐induced zeta potential change of LaCIONPs. f) In vitro release of La from LaCIONPs at different pH. g) In vitro release of iron ions from LaCIONPs at different pH. h) *In vitro* release profiles of CPT from LaCIONPs in the absence or presence of H_2_O_2_.

To demonstrate the pH‐triggered release behaviors of La and iron ions from LaCIONPs, the LaCIONPs were incubated with media at different pH values (7.4, 6.6, or 5.5). The amounts of released La and iron ions were measured by HPLC and inductively coupled plasma MS, respectively. As shown in Figure 2f, at pH 7.4 and pH 6.6, the LaCIONPs showed relatively slow La release rates; however, at pH 5.5, the release rate of La was significantly improved. The pH‐responsive release behavior of La is due to the pH‐induced disassembly of the LaCIONPs. Similarly, the pH values of media also played a decisive role in iron ion release (Figure 2g). After 24 h incubation, 4.2% of iron ions was released at pH 5.5, which was obvious higher than that at pH 7.4 and pH 6.6. This result could be attributed to the hydrophilic transition of PDPA chains in acidic environment and the following reaction between protons and IONPs. Although the release rate of iron ions was relatively low, the leached amount of iron ions was still enough to catalyze the Fenton reaction. The iron ions can react with H_2_O_2_ to produce ·OH through Fenton reaction, leading to degradation of methylene blue (Figure S15, Supporting Information). The H_2_O_2_‐responsive CPT release behavior of LaCIONPs was then investigated in vitro. As shown in Figure 2h, in the presence of H_2_O_2_, about 75% of CPT was released from LaCIONPs after 48 h incubation. This triggered CPT release behavior was due to the H_2_O_2_‐induced cleavage of peroxalate ester linker (Figure S16, Supporting Information). Therefore, the LaCIONPs can achieve H_2_O_2_‐responsive drug delivery, resulting in reduced side effects.

Then the La‐induced H_2_O_2_ generation was evaluated in NQO1‐overexpressing A549 cells. Cells were incubated with La at different concentrations for 2 h and the generated ROS inside cells was stained with an ROS probe, 2′,7′‐dichlorofluorescin diacetate (DCFH‐DA). After staining with Hoechst 33342, the cells were imaged by fluorescence microscopy. As shown in Figure S17, Supporting Information, green fluorescence was observed in La‐treated cells, demonstrated that the DCFH‐DA was oxidized into fluorescent dichlorofluorescein (DCF) by ROS inside cells. As the La concentration increased, the DCF staining efficiency improved, indicating higher ROS level. In contrast, the cells without La treatment showed negligible fluorescence, indicating relatively low ROS level. This La‐induced ROS generation was also confirmed by flow cytometry (FCM) analysis (**Figure** **3**a). To demonstrate that the ROS generated by La is H_2_O_2_, Catalase was used as the H_2_O_2_ scavenger. As shown in Figure S18, Supporting Information, when Catalase was added into the culture media, the intracellular ROS level was reduced. These results demonstrated that La can generate H_2_O_2_ in A549 cells. To demonstrate tumor‐specific H_2_O_2_ level amplification, 293T cell line with low NQO1 expression was used as a control. As shown in the FCM analysis result (Figure S19, Supporting Information), no difference in DCF staining was found between the control group and the La‐treated group, suggesting the critical importance of NQO1 in H_2_O_2_ generation.

**Figure 3:**
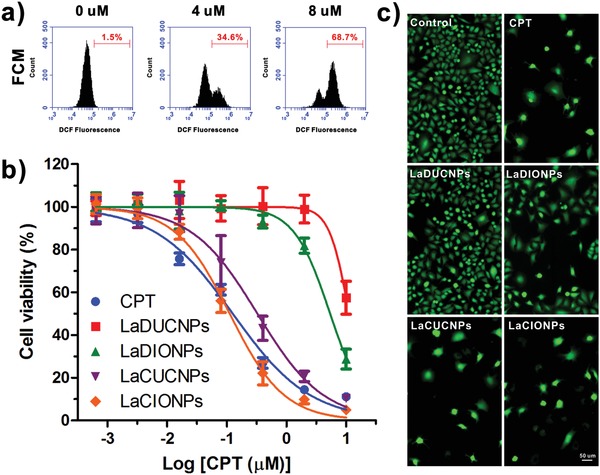
a) FCM analysis of A549 cells incubated with La at different concentrations for 2 h. Cells were stained with DCFH‐DA. b) Relative viability of A549 cells incubated with free CPT, LaDUCNPs, LaDIONPs, LaCUCNPs, or LaCIONPs for 48 h. The molar ratio of La to CPT was 1:5. c) Fluorescence images of A549 cells stained with Calcein AM after incubation with free CPT, LaDUCNPs, LaDIONPs, LaCUCNPs, or LaCIONPs for 48 h (La concentration: 0.4 µm; CPT concentration: 2 µm).

To investigate the antitumor activities of free CPT and nanomedicines in vitro, A549 cells were used. The cytotoxicity of DUCNPs and DIONPs without drugs was first assessed by the methyl thiazolyl tetrazolium assay. After 48 h of incubation, neither DUCNPs nor DIONPs showed obvious cytotoxicity to A549 cells (Figure S20, Supporting Information). However, when La was added to the nanosystems, the LaDUCNPs and LaDIONPs showed antitumor activities at high La concentrations (Figure 3b). The antitumor activity of LaDIONPs was higher than that of LaDUCNPs at same La concentration. This is because the released iron ions from IONPs converted less reactive H_2_O_2_ into highly toxic ·OH. In contrast, both LaDUCNPs and LaDIONPs showed low cytotoxicity to 293T cells (Figure S21, Supporting Information). Compared to LaDUCNPs and LaDIONPs, the polyprodrug‐containing nanomedicines (LaCUCNPs and LaCIONPs) showed effective antitumor activities due to the H_2_O_2_–triggered CPT release from nanomedicines. Importantly, compared to the monotherapy, the chemo/chemodynamic combination therapy showed the best antitumor effect, which was comparable to the highly potent free CPT. After different treatments, calcein AM was used for live cell staining. As shown in Figure 3c, compared to the control group, the nanomedicines treated groups showed fewer live cells, suggesting antitumor effect of the nanomedicines.

The in vivo biodistribution of LaCIONPs was then studied by positron emission tomography (PET) imaging using ^64^Cu as the radiotracer. The ^64^Cu‐labeled LaCIONPs were intravenously injected into A549 tumor‐bearing nude mice and the decay‐correlated PET images were acquired at 1, 4, 24, and 48 h postinjection. As shown in **Figure** **4**a, the injected LaCIONPs can gradually accumulate into tumor tissue. Quantitative 3D region‐of‐interest analysis was used to determine the distribution of LaCIONPs in the heart, liver, and tumor (Figure 4b and Figure S22, Supporting Information). After intravenous injection, the concentration of LaCIONPs in the heart decreased over time. The tumor accumulation of LaCIONPs was measured to be 4.93 ± 0.56% of injected dose per gram of tissue (% ID g^−1^) at 24 h post‐injection. Tumors and major organs were collected for ex vivo biodistribution study by using a γ‐counter at 48 h post‐injection. As shown in Figure 4c, the result was in good agreement with that derived from the PET images.

**Figure 4:**
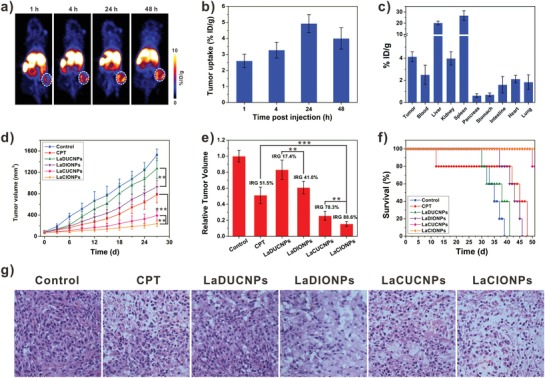
a) Whole‐body coronal PET images of A549 tumor‐bearing mice intravenously injected with ^64^Cu‐labeled LaCIONPs. White circles indicate tumors. b) Time‐dependent tumor uptake of ^64^Cu‐labeled LaCIONPs. c) Biodistribution of ^64^Cu‐labeled LaCIONPs at 48 h post‐injection. d) The growth curves of A549 tumor treated with saline, free CPT, LaDUCNPs, LaDIONPs, LaCUCNPs, or LaCIONPs (^**^ *p* < 0.01, ^***^ *p* < 0.001). e) The inhibition rate of tumor growth (IRG) at the end of treatment (^**^ *p* < 0.01, ^***^ *p* < 0.001). f) Survival curves of the mice after different treatments. g) Hematoxylin and eosin staining of tumors after different treatments.

Then the in vivo tumor therapy was evaluated on A549 tumor mice. When the tumors reached around 80 mm^3^, mice were intravenously injected with saline, free CPT, LaDUCNPs, LaDIONPs, LaCUCNPs, or LaCIONPs every 3 days for five times (CPT: 3 mg kg^−1^). Compared to saline‐treated control group, tumor growth suppressions were achieved in free CPT and nanomedicine treated groups (Figure 4d). Importantly, the chemo/chemodynamic combination therapy group showed much better antitumor effect than the other groups. On day 27, the relative tumor volumes and inhibition rates of tumor growth (IRG) of different groups were calculated. As shown in Figure 4e, the IRG of the LaCIONPs was 88.6%, which was much higher than those of free CPT (51.5%), LaDUCNPs (17.4%), LaDIONPs (41.0%), and LaCUCNPs (78.3%). Due to the great antitumor effect of chemo/chemodynamic combination therapy, the LaCIONP‐treated mice showed greatly prolonged survival time (Figure 4f). The hematoxylin and eosin staining and terminal deoxynucleotidyl transferase dUTP nick end labeling results of tumor tissue sections further confirmed the combination therapy induced tumor cell apoptosis (Figure 4g and Figure S23, Supporting Information). Importantly, during the treatment of nanomedicines, no severe body weight loss or major organ tissue damages of mice were observed, indicating low systemic toxicity of the nanomedicines (Figures S24 and S25, Supporting Information). In contrast, during the treatment with free CPT, obvious weight loss was observed, indicating systemic toxicity caused by free CPT.

In summary, a nanomedicine (LaCIONPs) that can achieve tumor‐specific drug release and ROS generation was developed for cancer chemo/chemodynamic therapy. The as‐prepared LaCIONPs showed nanosized diameter and PEG coating, resulting in tumor accumulation through EPR effect. Once the LaCIONPs enter cancer cells, their structure will be disintegrated in acidic intracellular environment, leading to rapid release of La and iron ions. Through tumor specific NAD(P)H: NQO1 catalysis, La can generate massive H_2_O_2_. On the one hand, H_2_O_2_ can further react with iron ions to produce highly toxic ·OH for chemodynamic therapy. On the other hand, H_2_O_2_ can also activate the CPT release from the polyprodrug for chemotherapy. Owing to the low NQO1 expression in normal cells, the LaCIONPs will not cause significant H_2_O_2_ level amplification and severe side effects in normal tissues. Therefore, this nanomedicine is a promising agent for tumor‐specific chemo/chemodynamic combination therapy.

## Conflict of Interest

The authors declare no conflict of interest.

## Supporting information

Supplementary
