# Supplementary material for: Tumor‐Specific Drug Release and Reactive Oxygen Species Generation for Cancer Chemo/Chemodynamic Combination Therapy
Source: Adv Sci (Weinh). 2019 Jan 18;6(5):1801986. doi: 10.1002/advs.201801986 (PMC6402284; doi:10.1002/advs.201801986)
Supplement: Supplementary file 1 — Supplementary [file ADVS-6-1801986-s001.pdf]

Copyright WILEY-VCH Verlag GmbH & Co. KGaA, 69469 Weinheim, Germany, 2019.

ADVANCED SCIENCE
Open Access

Supporting Information

for *Adv. Sci.,* DOI: 10.1002/advs.201801986

Tumor-Specific Drug Release and Reactive Oxygen Species Generation for Cancer Chemo/Chemodynamic Combination Therapy

Sheng Wang, Zhantong Wang, Guocan Yu, Zijian Zhou, Orit Jacobson, Yijing Liu, Ying Ma, Fuwu Zhang, Zhi-Yi Chen, and Xiaoyuan Chen**

Supporting Information

Tumor-Specific Drug Release and Reactive Oxygen Species Generation for Cancer Chemo/Chemodynamic Combination Therapy

Sheng Wang, Zhantong Wang, Guocan Yu, Zijian Zhou, Orit Jacobson, Yijing Liu, Ying Ma, Fuwu Zhang, Zhi-Yi Chen and Xiaoyuan Chen**

Materials

Poly(ethylene glycol) ($mPEG_{5k}$-NH_2) was purchased from Biochempeg (USA). 4-Cyano-4-(phenylcarbonothioylthio)pentanoic acid N-succinimidyl ester (RAFT-NHS), 2-bromoisobutyryl bromide, 2-(diisopropylamino)ethyl methacrylate (DPA), 2,2'-Dipyridyl (bpy), 2-hydroxyethyl methacrylate (HEMA), oxalyl chloride, bromotrimethylsilane, 2,2′-azobis(2-methylpropionitrile) (AIBN), 2′,7′-dichlorofluorescin diacetate (DCFH-DA), methylene blue (MB) and methyl thiazolyl tetrazolium (MTT) were obtained from Sigma-Aldrich. Dimethyl(2-hydroxyethyl) phosphonate was purchased from TCI. (S)-(+)-Camptothecin (CPT) was purchased from Chem-Impex (USA). β-Lapachone (La) was purchased from ApexBio (USA). The iron oxide nanoparticles (IONPs) and upconversion nanoparticles (UCNPs) were prepared according previous reports.[1]

Synthesis of CPTMA monomer

The CPTMA was synthesized according previous report (Scheme S1).[2] Briefly, HEMA (40 mmol) was dissloved in anhydrous dichloromethane (30 mL). Then oxalyl chloride (60 mmol) in anhydrous dichloromethane (50 mL) was dropwise added into the HEMA solution at 0 °C. After 1 h of reaction, the excess oxalyl chloride and solvent were removed under reduced pressure to afford a colorless oil. Subsequently, CPT (5.75 mmol) and triethylamine (9.1 mmol) in anhydrous dichloromethane (50 mL) was added into the prepared colorless oil

solution (9.1 mmol). After 1 h of reaction, the mixture was filtered and the solvent was evaporated under reduced pressure. Then the concentrated mixture was allowed to afford chromatographic separation (silica gel, dichloromethane/ethyl acetate (2:1, v/v)) to yield CPTMA prodrug monomer as a pale yellow solid.

Synthesis of PEG-PCPT

The PEG-PCPT was synthesized by a Reversible Addition–Fragmentation chain Transfer (RAFT) polymerization method (Scheme S2).[2] At first, a PEG-RAFT agent was synthesized by the reaction between $mPEG_{5k}$-NH_2 and RAFT-NHS agent. Then PEG-RAFT (0.02 mmol), CPTMA (1 mmol), AIBN (0.004 mmol) were dissolved in a mixture of 1, 4-dioxane (2 mL) and DMSO (2 mL) and added into a flask. The flask was sealed under dry argon and the polymerization was carried out at 80 °C for 24 h. The mixture was precipitated into an excess of ethyl acetate to generate PEG-PCPT.

Synthesis of PEG-PDPA

The PEG-PDPA was synthesized by RAFT polymerization (Scheme S3). PEG-RAFT (0.1 mmol), DPA (6 mmol), AIBN (0.02 mmol) were dissolved in 1, 4-dioxane (5 mL) and added into a flask. The flask was sealed under dry argon and the polymerization was carried out at 70 °C for 24 h. The product was dialyzed in pure water and lyophilized.

Synthesis of Dimethyl 2-(2-Bromoisobutyryloxy)ethyl) phosphonate (DMBiBEP) and 2-(2-Bromoisobutyryloxy)ethyl) phosphonic acid (BiBEP)

The DMBiBEP and BiBEP were synthesized according previous report (Scheme S4).[3] In brief, dimethyl(2-hydroxyethyl) phosphonate (10 g), triethylamine (10.3 mL) were dissloved in dichloromethane (120 mL). Then 2-bromoisobutyryl bromide (8.9 mL) was slowly added to the mixture at 0 °C. The reaction was held at room temperature for 12 h. The mixture was washed with saturated brine (2 × 150 mL) and water (1 × 100 mL). The organic layer was

separated and dried over anhydrous $MgSO_4$. The DMBiBEP was obtained after filtration and evaporation of the solvent.

To synthesize BiBEP, DMBiBEP (0.5 g) was dissloved in dichloromethane (5 mL). Bromotrimethylsilane (1.3 mL) was added in a dropwise manner, and the mixture was stirred at room temperature for 12 h. After evaporation of the solvent, methanol (5 mL) was added and the mixture was stirred at room temperature for another 12 h. The solvent was evaporated and the BiBEP was obtained after washing with ether.

Synthesis of phosphonated poly DPA (P-PDPA)

The P-PDPA was synthesized by atom transfer radical polymerization (ATRP) (Scheme S5). Briefly, DPA (1.07 g), bpy (50 mg) and BiBEP (27.5 mg) were charged into a polymerization tube. Then a mixture of 2-propanol (2 mL) and N,N-Dimethylformamide (DMF, 2 mL) was added to dissolve the monomer and initiator. After three cycles of freeze-pump-thaw to remove oxygen, CuBr (14.4 mg) was added into the reaction tube under nitrogen atmosphere. The polymerization was carried out at 40 °C for 24 h. After polymerization, the reaction mixture was diluted with tetrahydrofuran (THF) (15 mL), and passed through an Al_2O_3 column to remove the catalyst. Then THF was removed by rotovap. The mixture was precipitated into methanol/water to generate P-PDPA.

Preparation of PDPA-IONPs and PDPA-UCNPs

The PDPA-IONPs were prepared as follows: P-PDPA (50 mg) was dissolved in THF (5 mL) and then added into IONPs THF (5 mL, 1mg mL^{-1}). The above mixture was heated to 50 °C and incubated for 5 h under nitrogen atmosphere. The PDPA-IONPs was collected by centrifugation and redispersed in THF. The PDPA-UCNPs was synthesized under the same experimental conditions except that the IONPs were replaced with UCNPs.

Preparation of LaCIONPs

The LaCIONPs were prepared as follows: PEG-PCPT (1 mg), PEG-PDPA (3 mg), La (75 μg) and PDPA-IONPs (Fe content: 1 mg) were dissolved in dichloromethane (0.5 mL). The solution was added into distilled water (4 mL) dropwise under sonication. Then the organic solvent was then removed by rotary evaporation to form LaCIONPs suspension. The LaCUCNPs, LaDIONPs and LaDUCNPs were prepared under the similar experimental conditions.

Table S1. Sample compositions and their acronyms

Samples	La	PEG-PDPA	PEG-PCPT	PDPA-UCNPs	PDPA-IONPs
LaCIONPs	+	+	+	-	+
LaCUCNPs	+	+	+	+	-
LaDIONPs	+	+	-	-	+
LaDUCNPs	+	+	-	+	-

Characterizations

The morphology of the HRNMs was observed by Tecnai TF30 transmission electron microscope (TEM) (FEI, Hillsboro, OR). The effective particle diameters and zeta potential of the samples were determined by a SZ-100 nano particle analyzer (HORIBA Scientific, USA) at room temperature. UV-vis-NIR absorption spectra were measured by Genesys 10S UV-Vis spectrophotometer (Thermo Scientific, Waltham, MA).

In vitro drug release

The in vitro drug release behaviors of the samples were evaluated by a dialysis method. The samples were dispered in 2 mL of media and added to dialysis bags (MWCO: 1000 Da) and placed in 20 mL of environmental media. At appropriate time points, 2 mL of the medium was taken out and replaced with the same amount of fresh medium. The amounts of the released La and CPT were measured by HPLC. The amount of the released Fe was measured by inductively coupled plasma mass spectrometry (ICP-MS).

In vitro cell experiments

The in vitro cell cytotoxicity, ROS generation study and antitumor activity study were assessed on A549 cell line, which was purchased from American type culture collection (ATCC).

To assess the cell cytotoxicity of DIONPs and DUCNPs, A549 cells were seeded into 96-well plates at a density of 3000 cells per well and incubated at 37 °C in 100 µL of DMEM with 10% FBS under a 5% CO_2 atmosphere for 24 h. Then the samples were added to each well and the cells were incubated at 37 °C for an additional 48 h. After the incubation, the relative cell viabilities were measured by the MTT assay.

To assess the intracellular ROS generation of La, A549 cells were seeded into 8-well plates and incubated with DCFH-DA (25 µM) and La at different concentrations for 2 h. Then the cell nucleus were stained with Hoechst. The stained cells were observed with a fluorescence microscope.

For in vitro antitumor activities, A549 cells were seeded into 96-well plates and incubated with samples for 48 h. Thereafter, the relative cell viabilities were measured by MTT assay.

For live cell staining, A549 cells were seeded into 24-well plates and incubated with samples for 48 h (CPT concentration: 2 µM; La concentration: 0.4 µM). Thereafter, the cells were stained with Calcein-AM and imaged by an Olympus inverted microscope.

Animal model

All animal experiments were performed under a National Institutes of Health Animal Care and Use Committee (NIHACUC) approved protocol. Athymic nude mice (Harlan, Indianapolis, IN) were subcutaneously implanted with 5×10^6 of A549 cells.

In vivo PET imaging

NOTA-PDPA was synthesized for ^{64}Cu labeling. The NOTA-PDPA was encapsulated into LaCIONPs during preparation and then labeled with ^{64}Cu, obtaining ^{64}Cu- LaCIONPs. Then

the sample solution (100 μL, 140 μCi) was intravenously injected into A549 tumor-bearing mice. An Inveon small-animal PET scanner (Siemens, Erlangen, Germany) was used for the scanning at indicated time points after injection. At 48 h post-injection, the mice were sacrificed and the major organs were collected and assayed for radioactivity using a gamma counter. The percent of injected dose/gram of tissue (%ID/g) was then calculated.

In vivo therapy

A549 tumor-bearing mice were randomly divided into 6 groups (n = 5): control group, free CPT group, LaDUCNPs group, LaDIONPs group, LaCUCNPs group and LaCIONPs group. When the tumors reached about 80 mm^3, the mice were treated with samples (3 mg CPT kg^{-1}) via intravenous injection every 3 days for 5 times. Tumor volume and body weight were monitored every 3 days. Tumor volume was calculated as (major axis) × $(\text{minor axis})^2/2$. Mice were euthanized when major axis of tumor exceeded 20 mm or when mouse weight lost by over 20%.

[1] a) Z. Zhou, J. Song, R. Tian, Z. Yang, G. Yu, L. Lin, G. Zhang, W. Fan, F. Zhang, G. Niu, L. Nie, X. Chen, Angew. Chem. Int. Ed. **2017**, 56, 6492; b) S. Wang, W. Yang, J. Cui, X. Li, Y. Dou, L. Su, J. Chang, H. Wang, B. Zhang, Biomater. Sci. **2016**, 4, 338.
[2] H. Dong, J. Huang, R. R. Koepsel, P. Ye, A. J. Russell, K. Matyjaszewski, Biomacromolecules **2011**, 12, 1305.
[3] J. Li, Y. Li, Y. Wang, W. Ke, W. Chen, W. Wang, Z. Ge, Nano Letters **2017**, 17, 6983.
[4] F. Zhang, G. Zhu, O. Jacobson, Y. Liu, K. Chen, G. Yu, Q. Ni, J. Fan, Z. Yang, F. Xu, X. Fu, Z. Wang, Y. Ma, G. Niu, X. Zhao, X. Chen, ACS nano **2017**, 11, 8838.

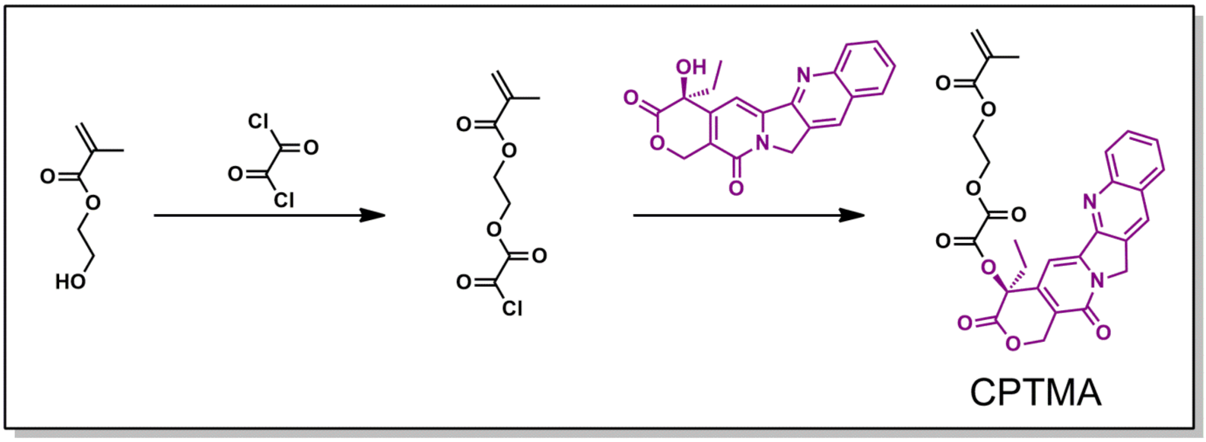

Scheme S1. Synthesis process of CPTMA monomer.

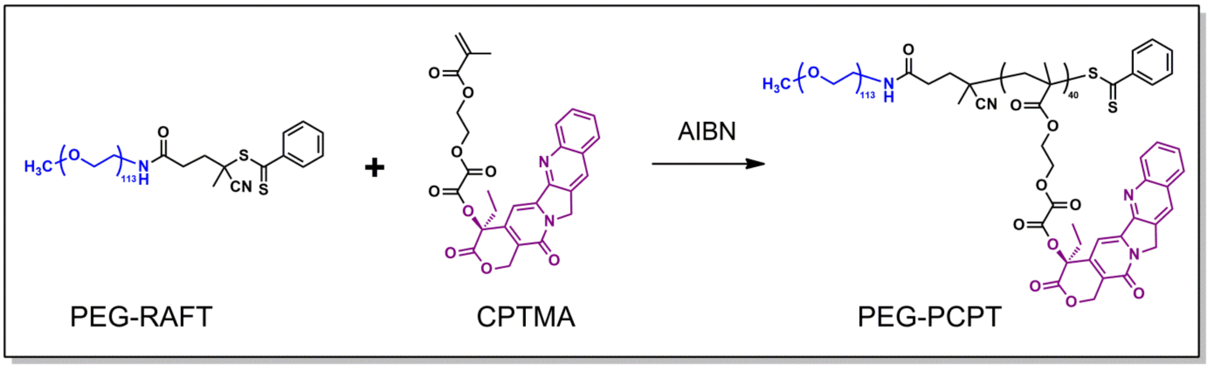

Scheme S2. Synthesis process of PEG-PCPT.

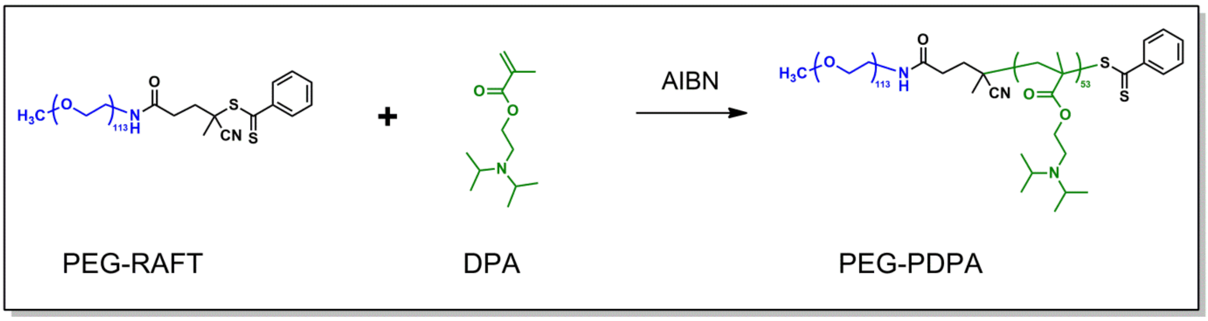

Scheme S3. Synthesis process of PEG-PDPA.

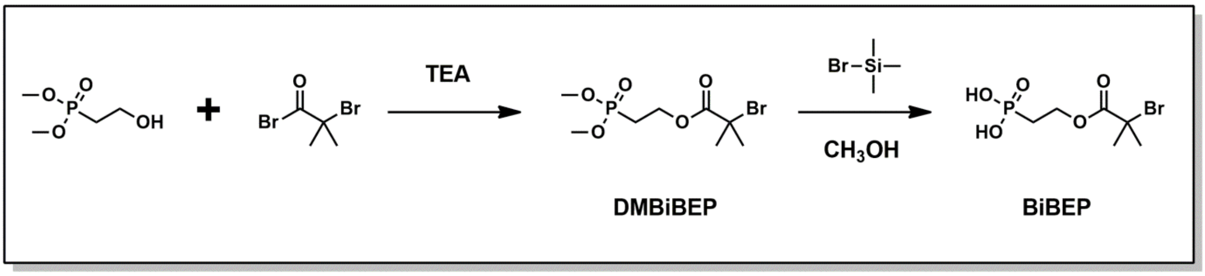

Scheme S4. Synthesis process of BiBEP.

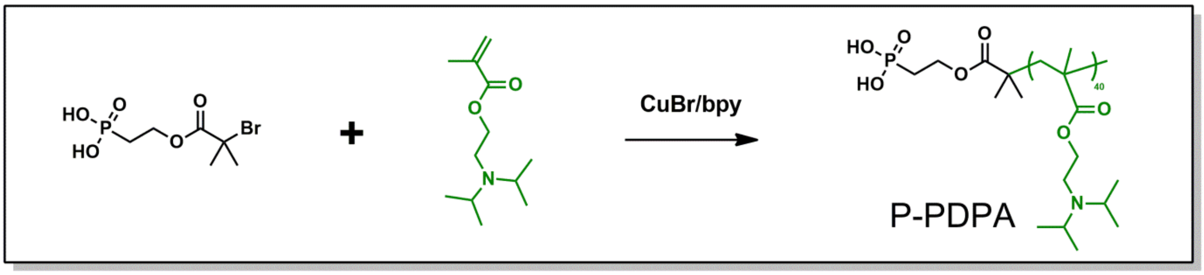

Scheme S5. Synthesis process of P-PDPA.

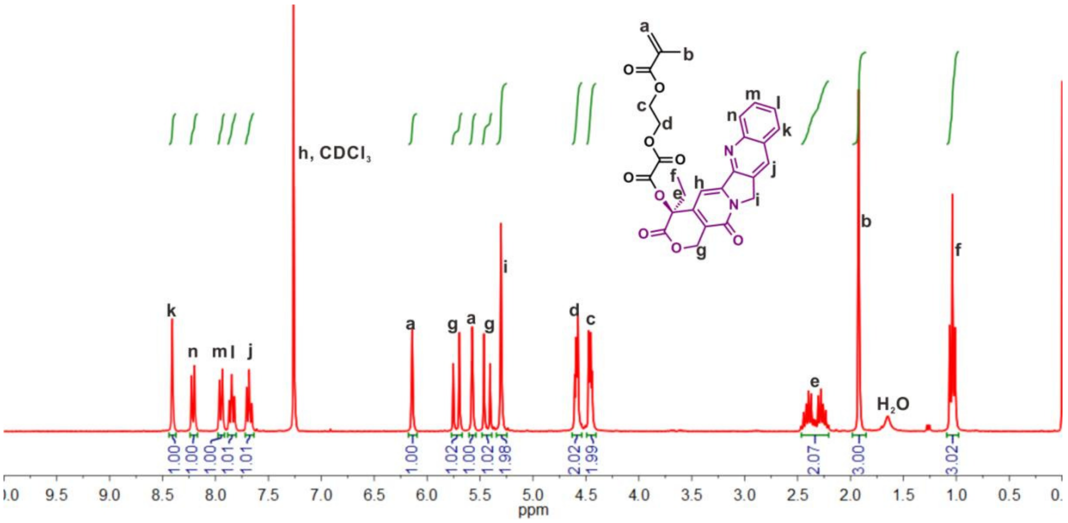

Figure S1. ^{1}H NMR spectrum of CPTMA.

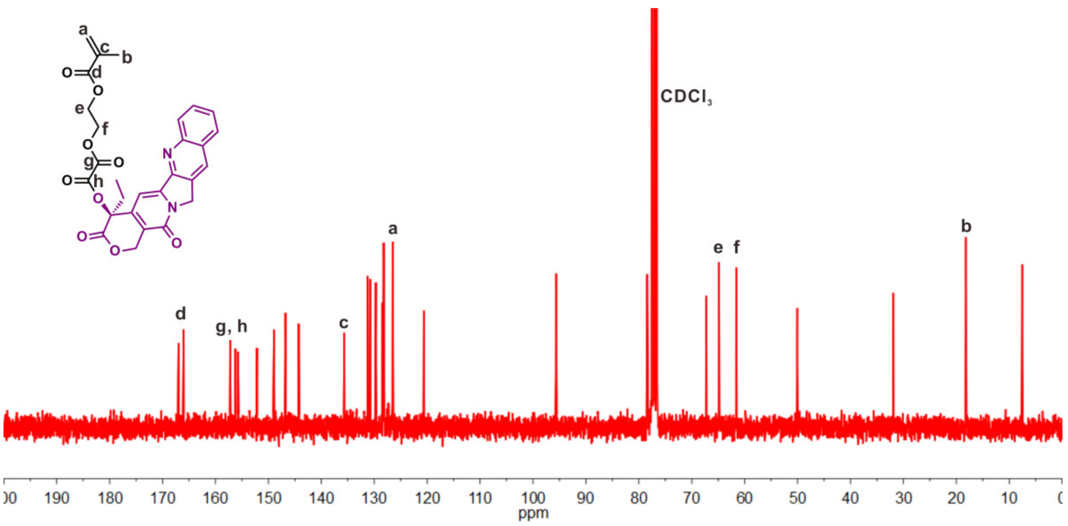

Figure S2. ^{13}C NMR spectrum of CPTMA.

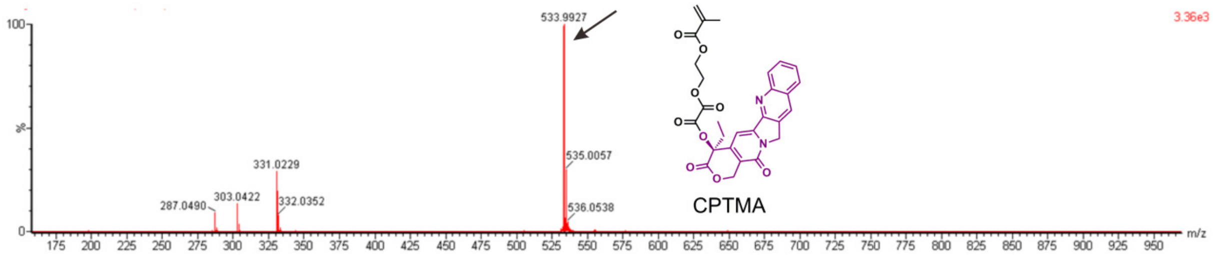

Figure S3. Liquid chromatography–mass spectrometry (LC-MS) analysis of CPTMA.

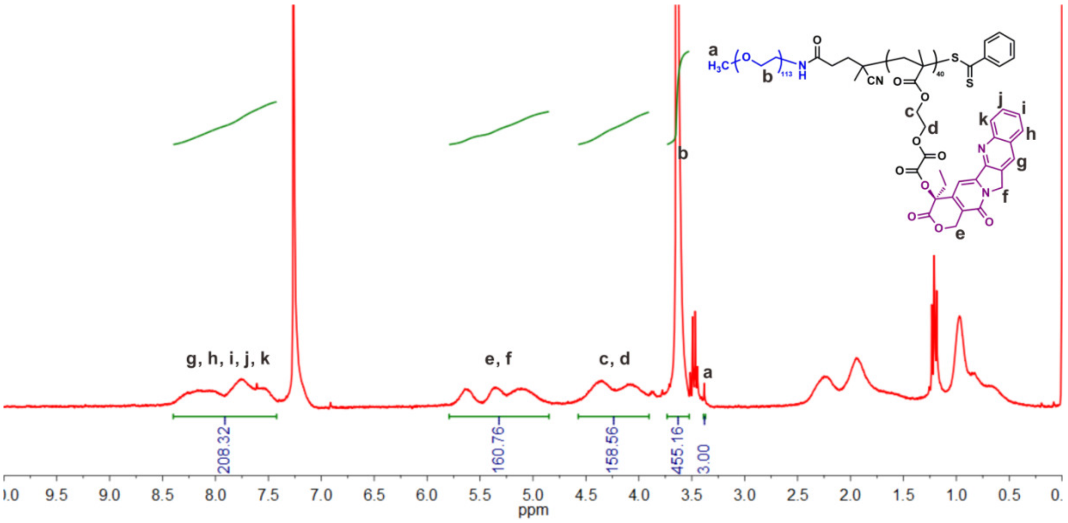

Figure S4. ^{1}H NMR spectrum of PEG-PCPT.

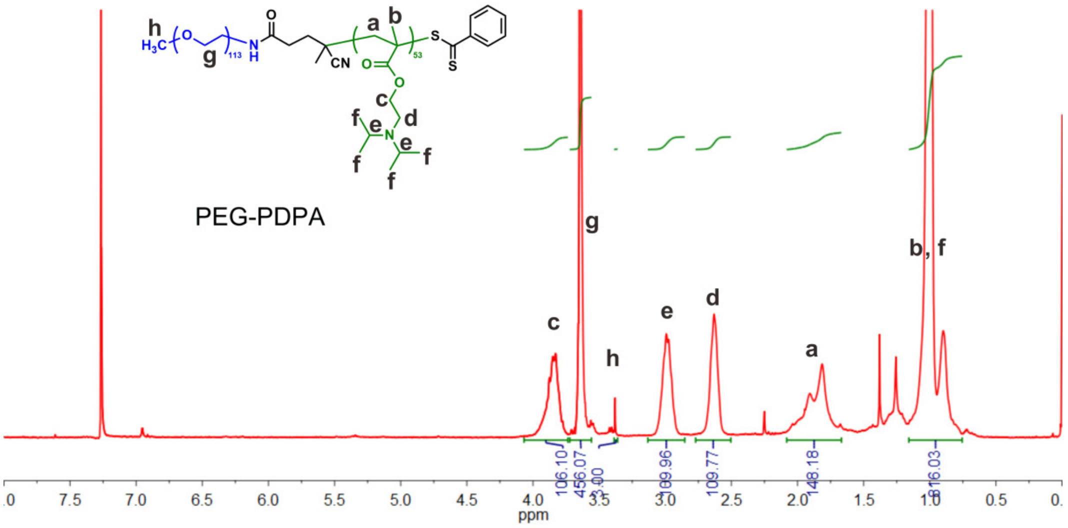

Figure S5. ^{1}H NMR spectrum of PEG-PDPA.

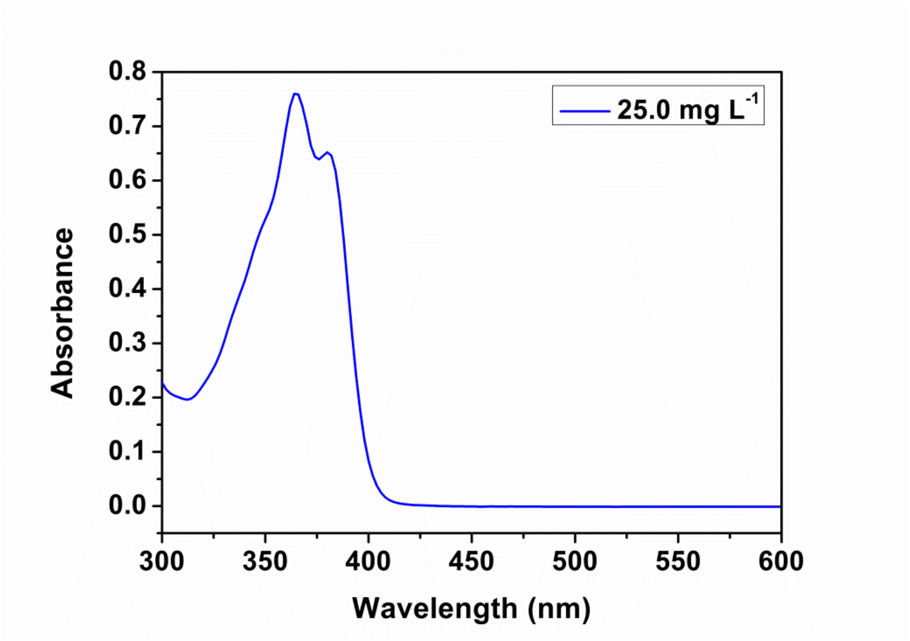

Figure S6. Absorption spectrum of PEG-PCPT in DMSO at a concentration of 25.0 mg L^{-1}.

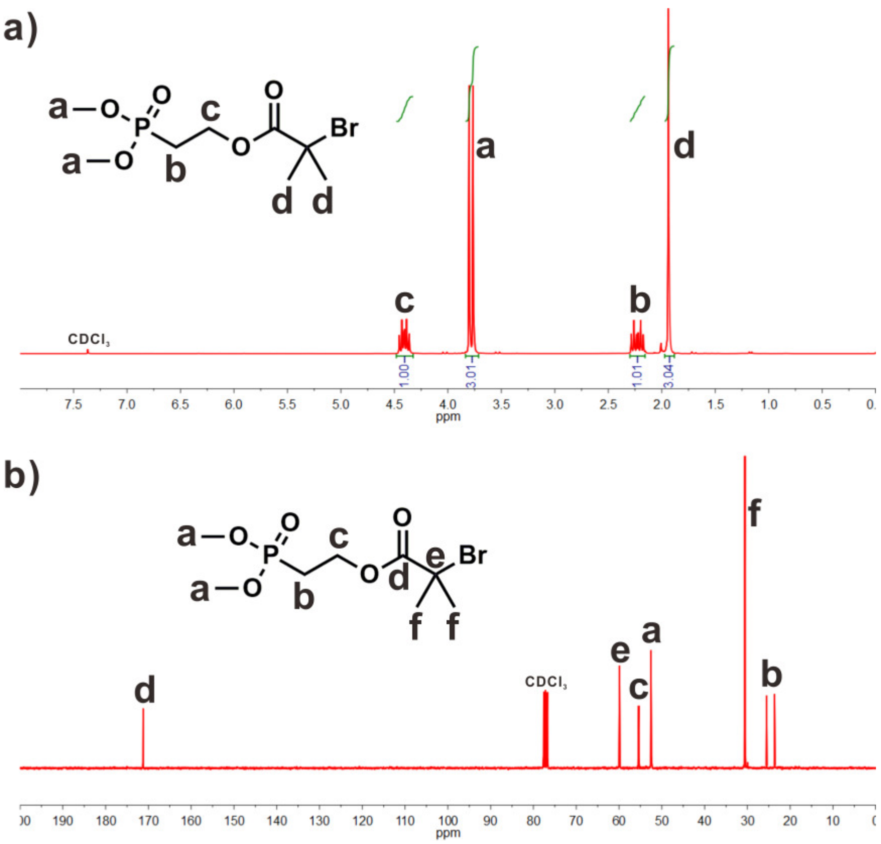

Figure S7. ^{1}H and ^{13}C NMR spectra of DMBiBEP.

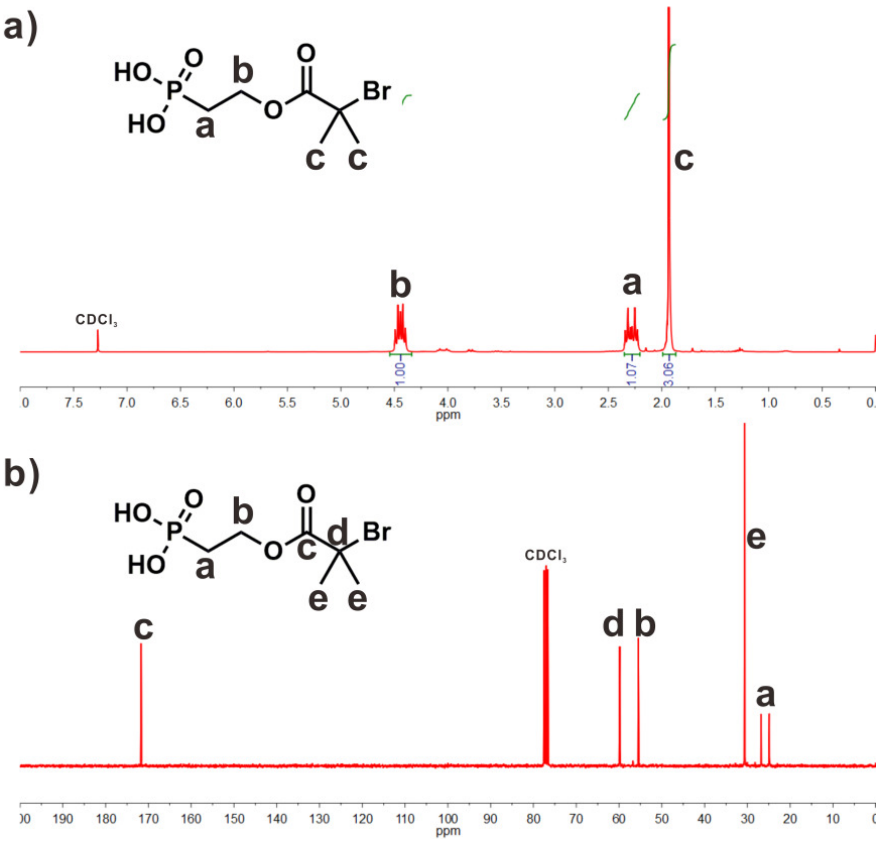

Figure S8. ^{1}H and ^{13}C NMR spectra of BiBEP.

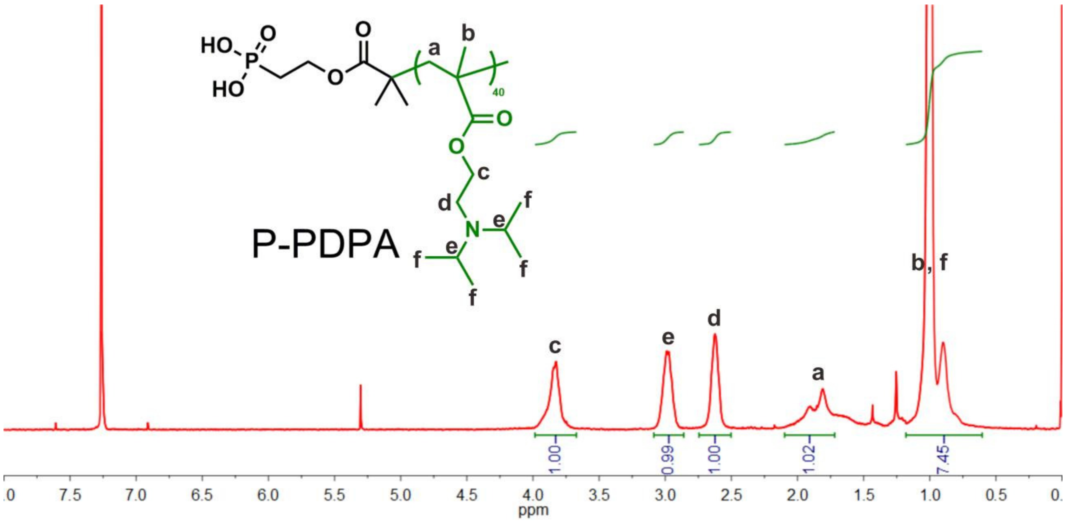

Figure S9. ^{1}H NMR spectrum of P-PDPA.

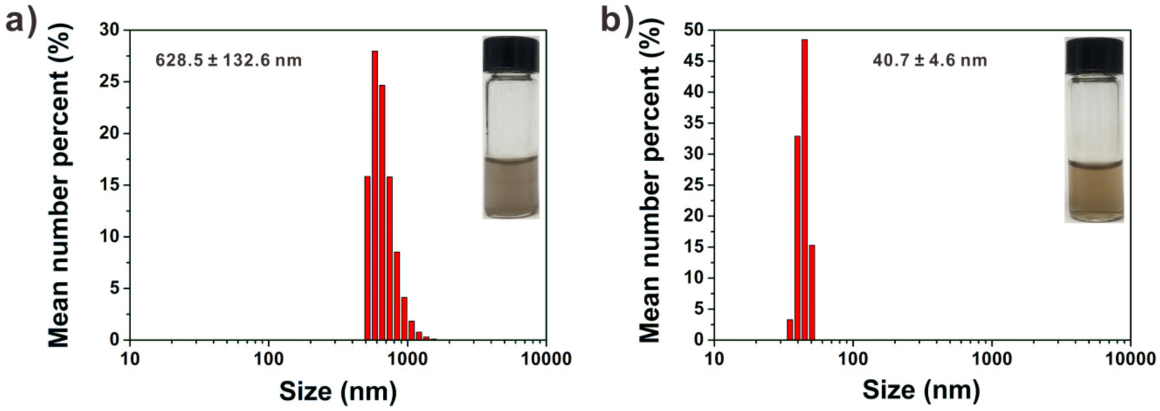

Figure S10. Particle diameters of PDPA-IONPs at pH 7.4 (a) and 5.5 (b).

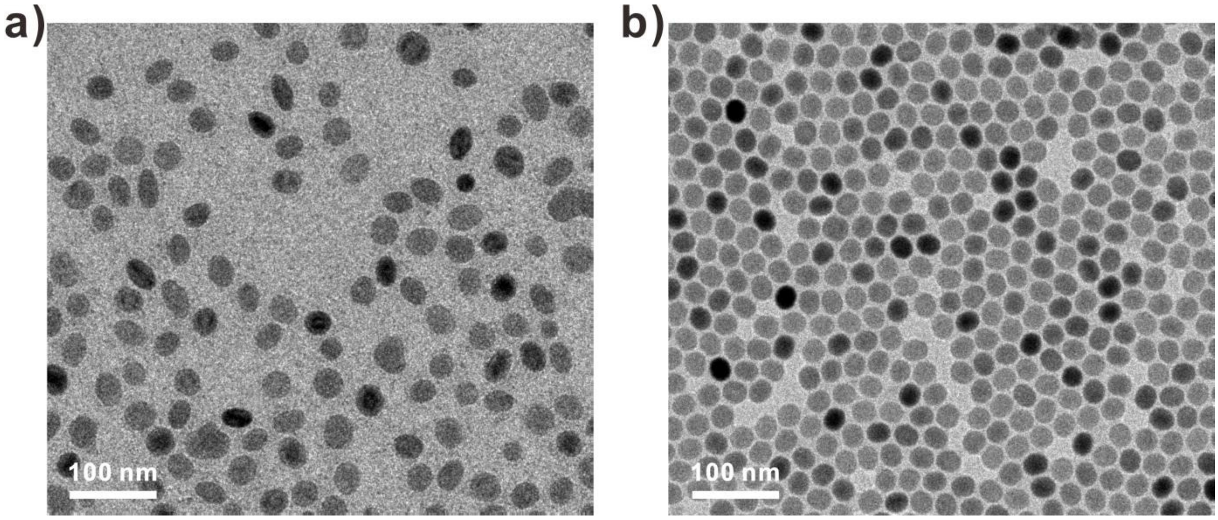

Figure S11. TEM images of PDPA-IONPs (a) and PDPA-UCNPs (b).

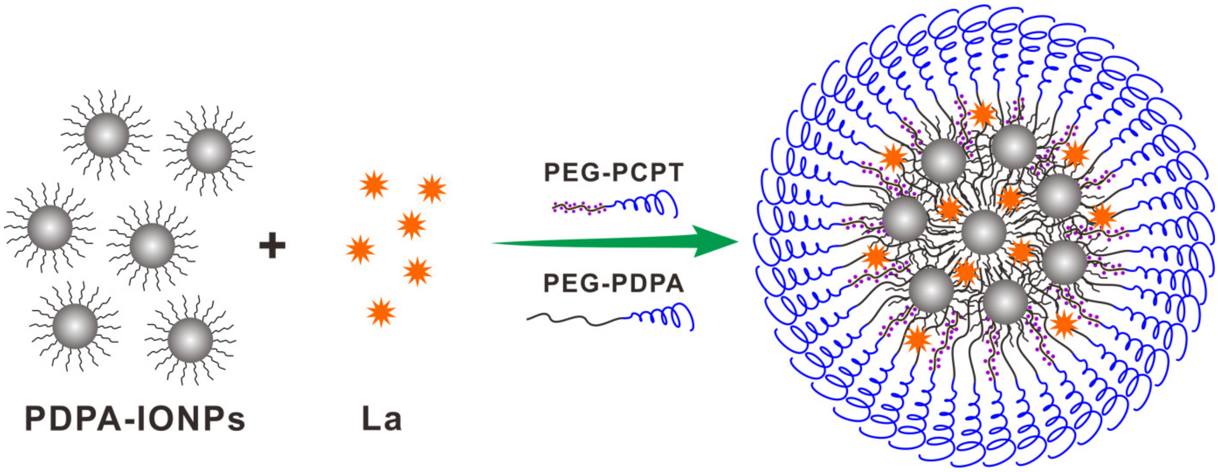

Figure S12. Schematic illustration showing the preparation process of LaCIONPs.

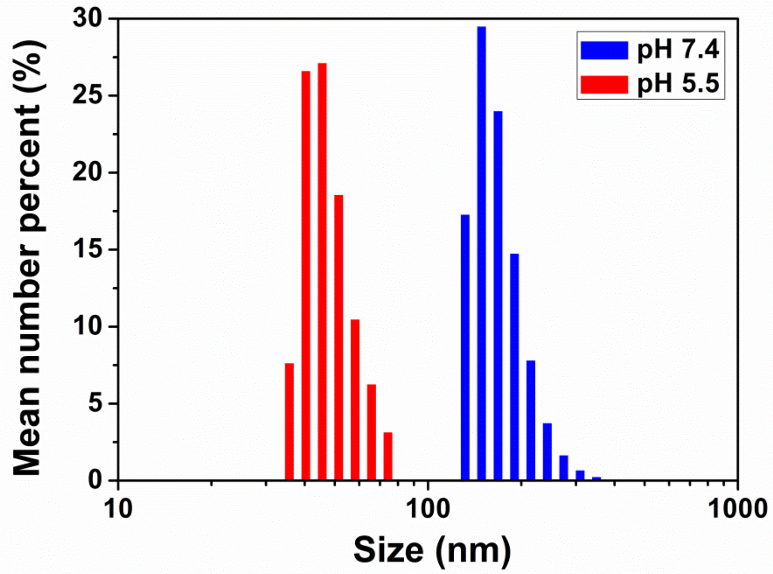

Figure S13. Hydrodynamic diameters of LaCUCNPs at different pH.

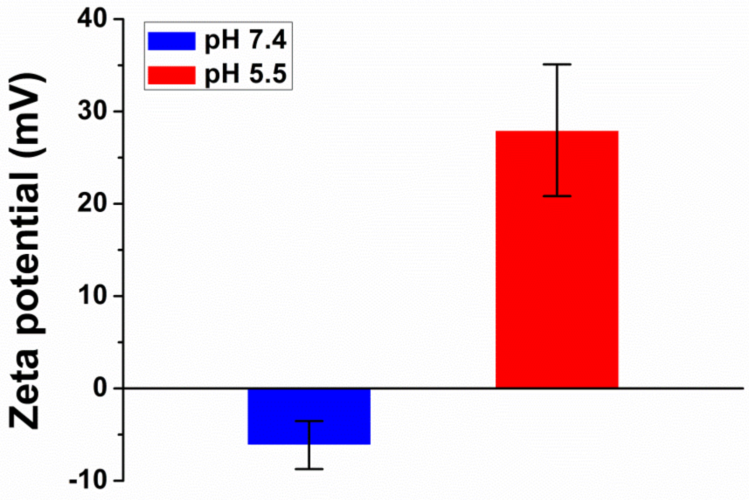

Figure S14. pH-induced zeta potential change of LaCUCNPs.

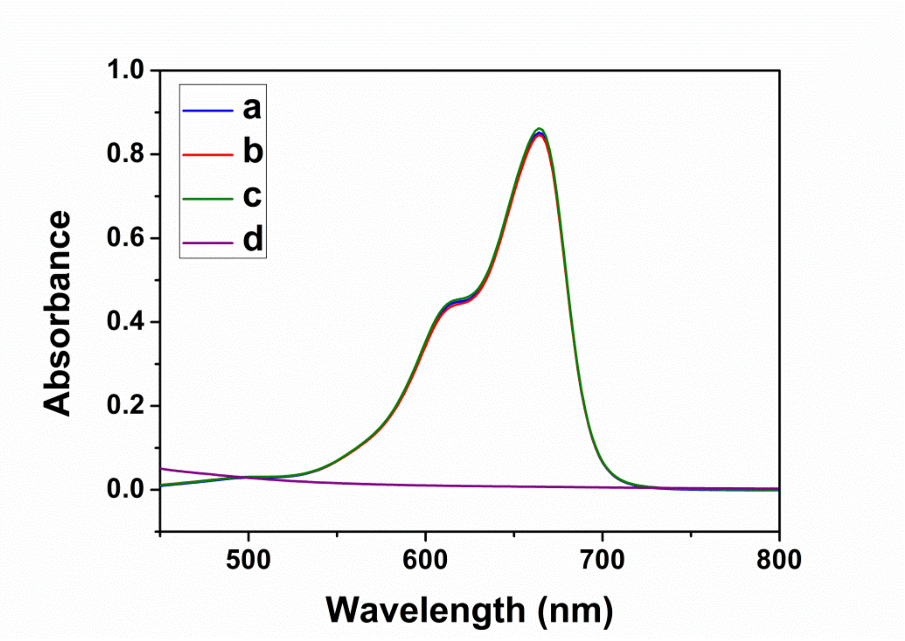

Figure S15. UV/Vis absorption spectra of MB after degradation by Fenton reaction. a) Pre and c) post treatment of H_2O_2 in the absence of iron ions. b) Pre and d) post treatment of H_2O_2 in the presence of iron ions.

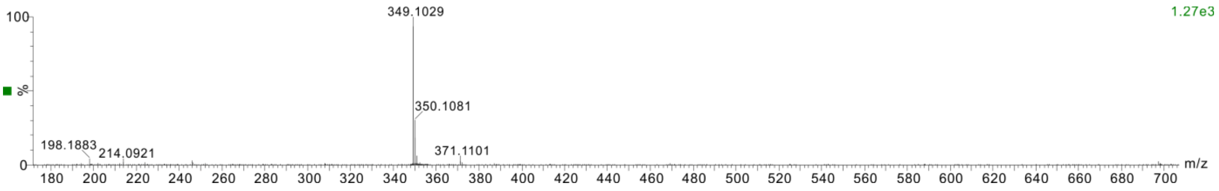

Figure S16. Mass spectrum of the released products from CIONPs with the treatment of 1 mM H_2O_2.

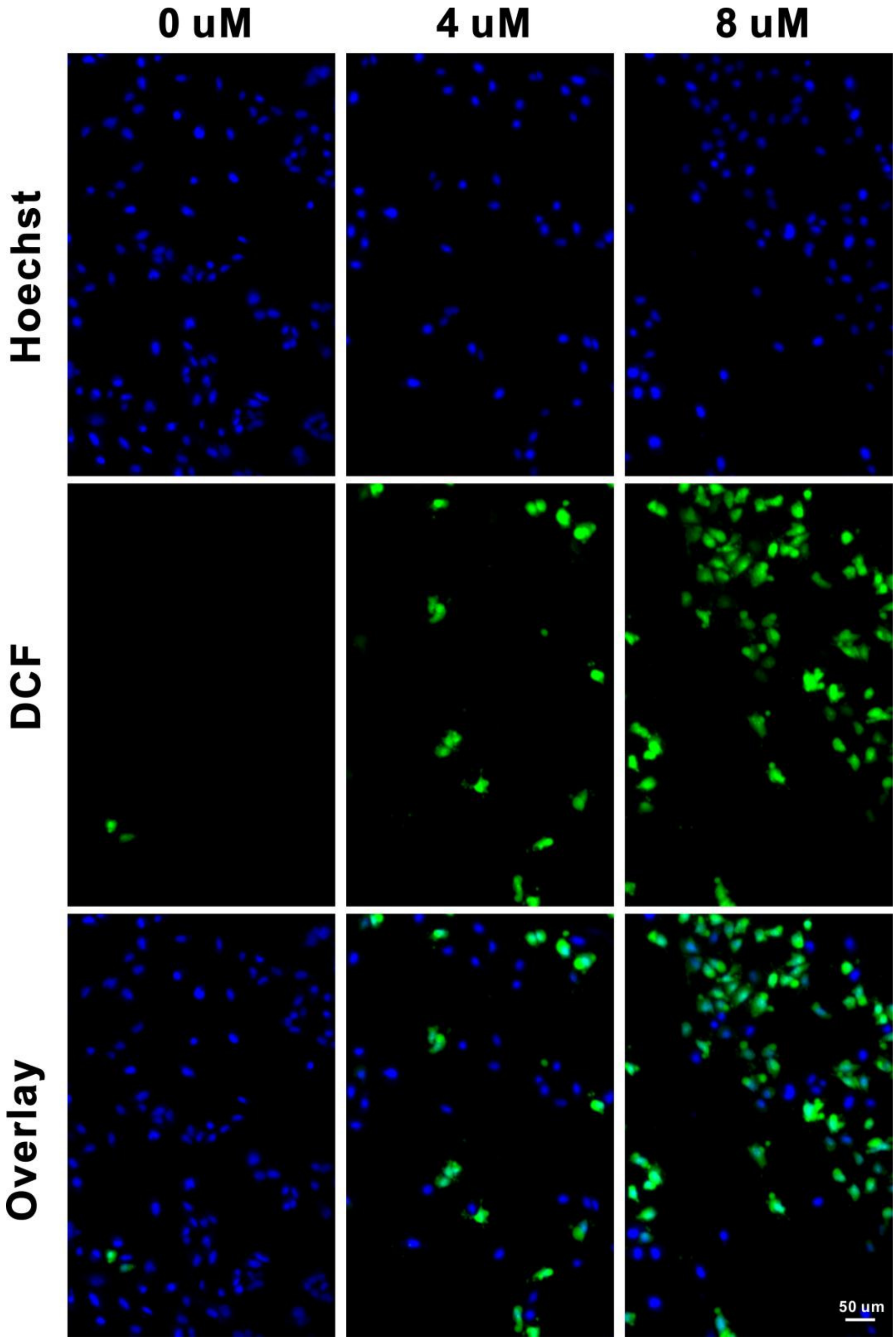

Figure S17. Fluorescence images of A549 cells incubated with La at different concentrations for 2 h. Cells were stained with Hoechst 33342 (blue) and DCFH-DA.

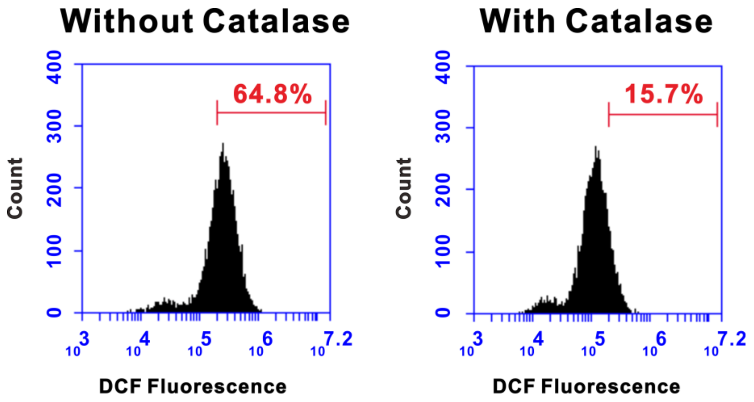

Figure S18. FCM analysis of A549 cells incubated with La (8×10^{-6} M) with or without Catalase for 2 h. H_2O_2 was stained with DCFH-DA.

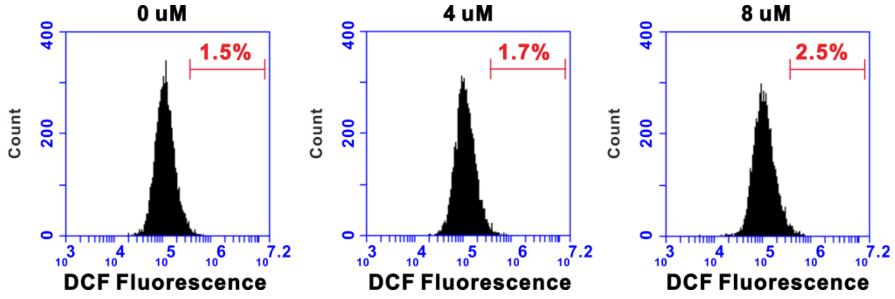

Figure S19. FCM analysis of 293T cells incubated with La at different concentrations for 2 h. H_2O_2 was stained with DCFH-DA.

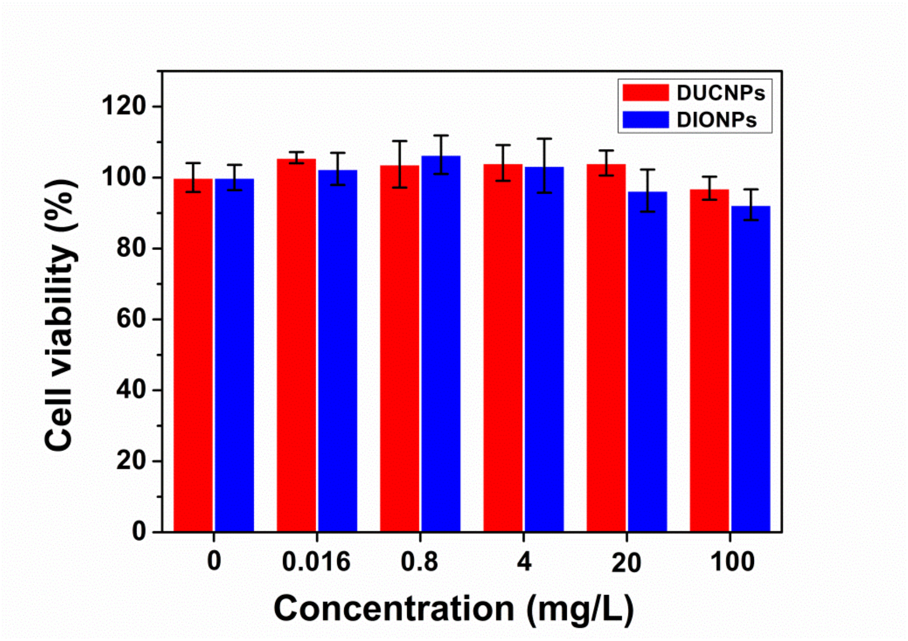

Figure S20. Relative viability of A549 cells incubated with DUCNPs and DIONPs for 48 h.

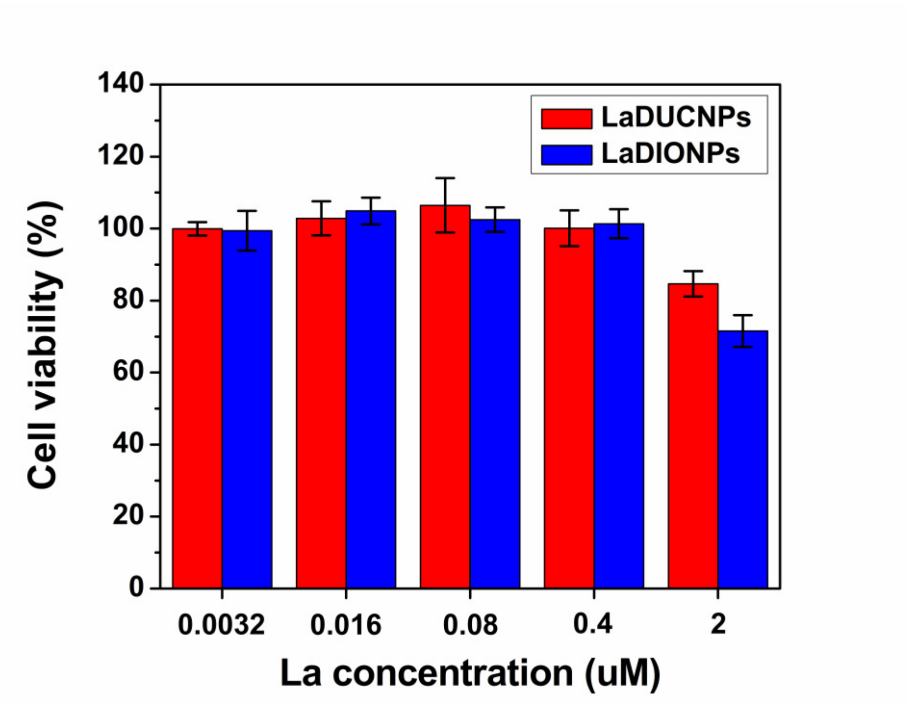

Figure S21. Relative viability of 293T cells incubated with LaDUCNPs and LaDIONPs for 48 h.

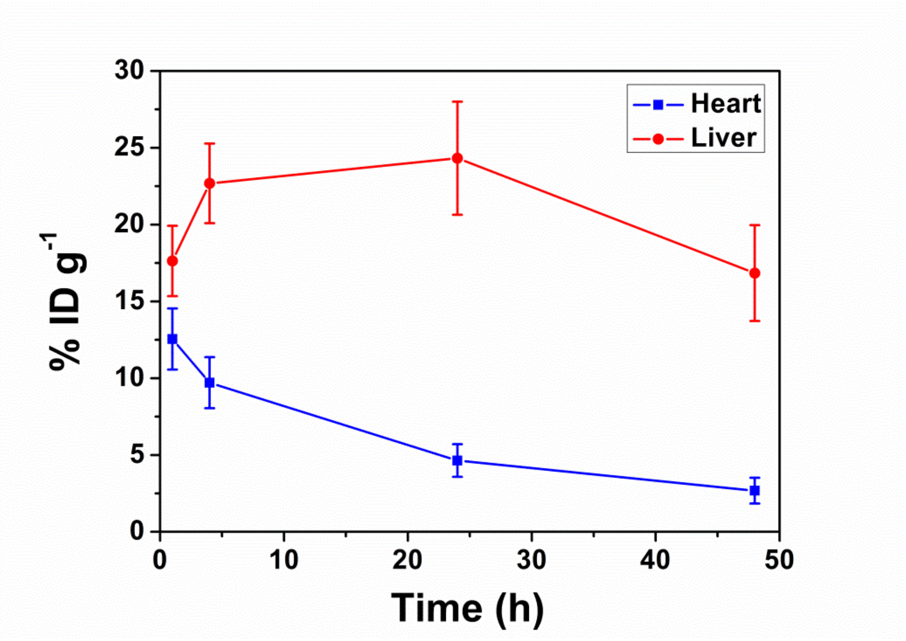

Figure S22. The distribution of LaCIONPs in heart (with blood) and liver at different time points after injection.

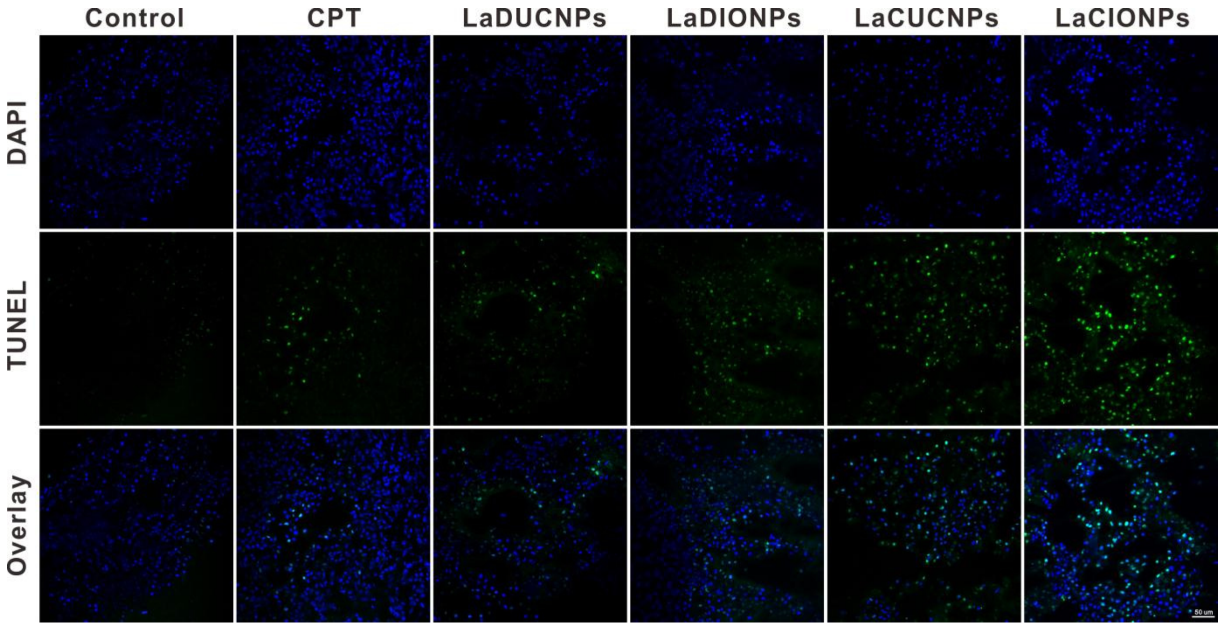

Figure S23. TUNEL analysis of tumor tissues after different treatments.

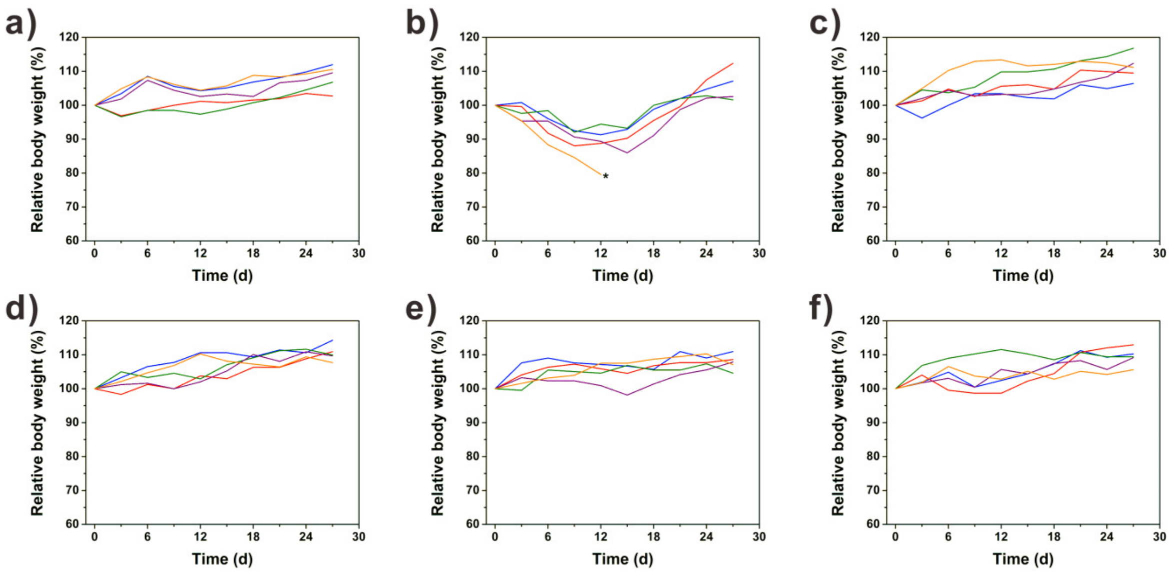

Figure S24. Mice body weight changes during the treatments. a) control, b) free CPT, c) LaDUCNPs, d) LaDIONPs, e) LaCUCNPs, f) LaCIONPs. *: the mouse was euthanized due to siginificant weight loss (over 20%).

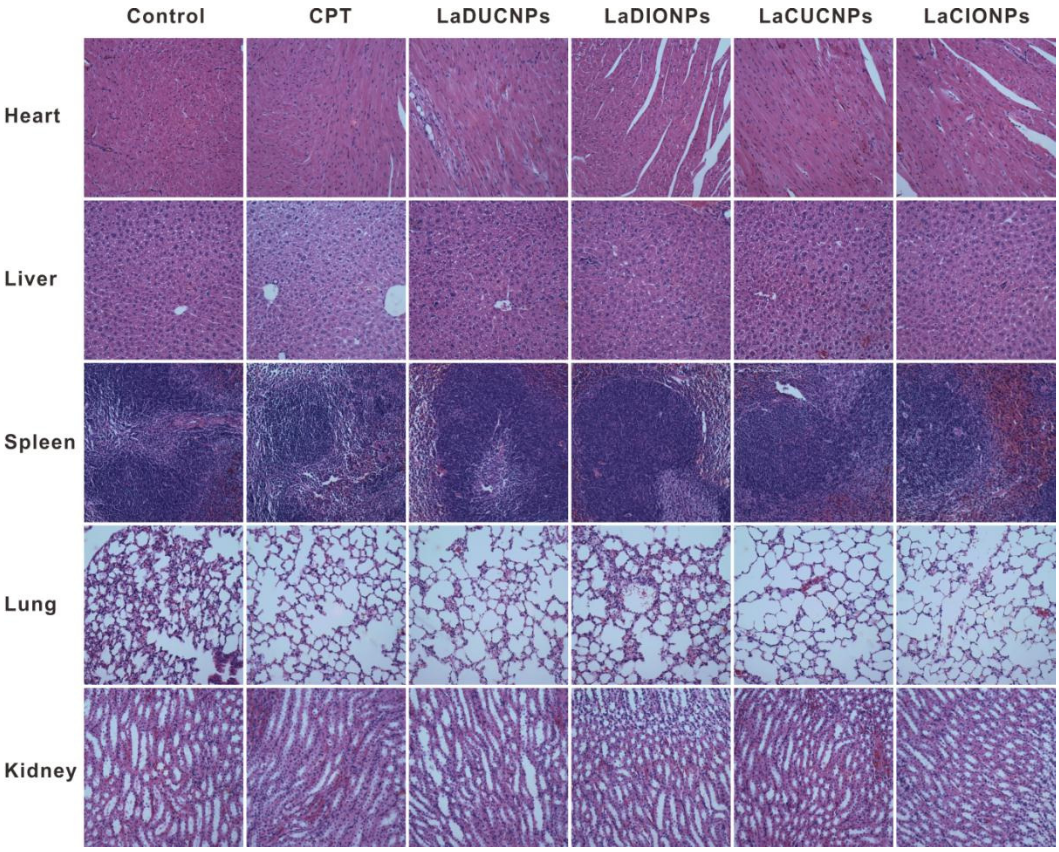

Figure S25. H&E staining of major organs.
